# Comparison of Contrast-Enhanced Ultrasonography to Color Doppler Ultrasound in Evaluation of Carotid Body Tumors

**DOI:** 10.3389/fonc.2022.872890

**Published:** 2022-04-11

**Authors:** Guangchao Gu, Xiaoyan Zhang, Junyue Shen, Shayan Gulidanna, Qiong Gao, Jiang Shao, Bao Liu, Bo Zhang, Yuehong Zheng

**Affiliations:** ^1^ Department of Vascular Surgery, Peking Union Medical College Hospital, Beijing, China; ^2^ Department of Diagnostic Ultrasound, Peking Union Medical College Hospital, Beijing, China; ^3^ School of Medicine, Tsinghua University, Beijing, China; ^4^ Key Laboratory of Carcinogenesis and Translational Research (Ministry of Education/Beijing), Department of Radiation Oncology, Peking University Cancer Hospital & Institute, Beijing, China; ^5^ Department of Ultrasound, China-Japan Friendship Hospital, Beijing, China; ^6^ State Key Laboratory of Complex Severe and Rare Diseases, Peking Union Medical College Hospital, Chinese Academy of Medical Science and Peking Union Medical College, Beijing, China

**Keywords:** carotid body tumor, contrast-enhanced ultrasound, time–intensity curve, morphology, vascularity, color Doppler ultrasound

## Abstract

**Objective:**

The objectives of this study were to prospectively 1) explore the characteristics and enhanced patterns of carotid body tumors (CBTs) at color Doppler ultrasound (CDU) and contrast-enhanced ultrasonography (CEUS) qualitatively and quantitatively and 2) compare CDU and CEUS for their morphology and vascularity signature.

**Methods:**

CDU and CEUS with Sonovue^®^ were used to evaluate 25 CBT lesions. The comparison between these ultrasonic modalities included the size, Shamblin type, vascularity, and feeding vessels of the lesion areas. The time–intensity curve (TIC) analysis was used to obtain the dynamics of the contrast-enhancement features of CBTs.

**Results:**

The TIC analysis presented a fast wash-in [wash-in time: 3.00 ± 1.10 s, mean ± SD] and slow wash-out [wash-out time: 58.79 ± 24.21 s, mean ± SD] pattern in the CBT lesions, with a high area under the curve (AUC) of 669.68 ± 143.46 mm^2^ (mean ± SD). In comparison with CDU, CEUS was superior in identifying Shamblin type I or III CBT lesions (χ^2 ^= 17.389, p=0.002). It detected a significant difference in the AUC between moderate and marked vascularity groups (563.33 ± 102.63 vs. 707.22 ± 138.81, t=-2.311, p=0.031.), while CDU observed no significant difference between these two groups. Although CDU was more sensitive than CEUS in detecting feeding vessels (100% vs. 88%), CEUS better visualized the origins of feeding vessels (χ^2 ^= 9.162, p=0.010).

**Conclusion:**

CEUS can better investigate the Shamblin type and vascularity of CBT lesions than CDU. CBTs displayed a fast wash-in, slow wash-out pattern with high AUC in the TIC analysis in the CEUS mode. CDU is more sensitive in detecting feeding vessels than CEUS, while CEUS can better visualize the origins of feeding vessels.

## Introduction

Carotid body tumors (CBTs) are non-chromaffin and slowly growing paragangliomas, located at the carotid artery bifurcation area. CBTs represent more than 50% of head and neck paragangliomas (1, 2), and 4%–6% of these neoplasms have a malignant tendency to develop distant metastasis (3). Surgical resection is the most common treatment for CBTs (4), and surgical treatment as early as possible is advisable.

Imaging modalities are essential for the diagnosis of CBTs. Currently, color Doppler ultrasound (CDU) is widely used in the diagnostic workup for CBTs due to the absence of radiation and its convenience. Identifying CBTs relies primarily on their specific location at carotid bifurcation, which leads to the splaying of carotid vessels, as well as the hyervascularity nature of CBTs (4). However, CDU was reported to have lower sensitivity in detecting CBTs (5), as well as poorer accuracy in characterizing the size of the lesion areas and Shamblin type of CBTs (6, 7) than computed tomography angiography (CTA). CTA, however, although widely accepted as the initial diagnostic tool of CBTs (4, 8), poses the risk of adverse effects, including exposure to ionizing radiation and nephrotoxicity from iodinated contrast material, and is unsuitable for vulnerable populations such as pediatric patients, pregnant patients, or those with chronic renal disease. Magnetic resonance angiography cannot be performed when a patient has a pacemaker or stainless stell prosthesis. Therefore, the utilization of new imaging modalities, such as contrast-enhanced ultrasonography (CEUS), has been considered as potentially beneficial to characterizing CBTs.

Studies have shown that CEUS using second-generation microbubble agents substantially improves the ability of CDU for detecting and characterizing lesion areas in several organs (e.g., liver, breast) (9) but not yet extensively in detecting CBTs. Sasan et al. demonstrated that CEUS could better identify liver lesions that were not visualized by CDU and promoted the ultrasound-guided liver biopsy (10). Moreover, time–intensity curve (TIC) analysis could quantitatively obtain the tumor perfusion feature and vascularity, which had been used in differential diagnosis (11). Other studies reported that CEUS was superior to CDU and equal to contrast-enhanced CT in assessing tumor vessels (12), which has great clinical significance to the preoperative evaluation of CBTs. Therefore, CEUS might be able to assess the morphology and vascularity of CBTs more accurately and provide a better preoperative assessment than CDU. However, the utilization and performance of CEUS on the evaluation of CBTs remain to be investigated.

This study aimed to prospectively explore the characteristics and enhanced patterns of CBTs at CDU and CEUS qualitatively and quantitatively and compare CDU and CEUS for their morphology and vascularity signature.

## Methods

### Patients

Ethical approval for the study was obtained from the institutional review board of Peking Union Medical College Hospital (PUMCH), and informed consent was obtained from all patients for participating in this study. From September 2017 to January 2018, 20 patients who were referred to our Vascular Center for further evaluation of CBTs were enrolled in the current study. The inclusion criteria were the presence of lateral neck masses and written informed consent; CTA performed and proved to be CBTs according to typical radiographic characteristics (4). The exclusion criteria were suspected allergy to Sonovue^®^, pregnancy or lactation, severe cardiac and respiratory dysfunctions, and lesion areas with previous embolization therapy. All patients underwent ultrasonic scanning, including CDU and CEUS, and CTA scanning for CBT lesions.

### Computed Tomography Angiography

All patients underwent CTA tests, either prior to admission or during hospitalization. The parameters of the CTA examinations in our institution were adequately described in a previous study (13). Briefly, a 192-slice CT system (SOMATOM Force) was used to examine the patients lying in supine. Iodixanol (370 mg of iodine per milliliter) was injected intravenously into either of the antecubital veins. The injection was administered through an auto-injector, and each patient received 40 ml of iodixanol and 50 ml of saline solution at a rate of 5 ml per second. Studies were performed with the semi-automated CARE kV with 120 kVp, and 84 mAs referred to the vendor’s recommendation. The bolus-tracking technique was used. A dedicated workstation (syngo. via VA30) was used to reconstruct the multiplanar reformation (MPR) and maximum intensity projection (MIP) images.

### Diagnosis and Shamblin Typing of Carotid Body Tumors

Neck lesions with the typical characteristics of the splaying of carotid bifurcation and hyervascularity nature shown on CTA were diagnosed as CBTs according to the diagnostic criteria from literature (4). Shamblin type of CBT lesions was defined by radiographic findings according to the literature (7, 8), whereby type I, II, and III lesions indicate tumors with no encasement, partial encasement, and complete encasement to carotid arteries, respectively.

### Ultrasound Scanning

CDU was performed by an experienced examiner with an IU22 scanner (Phillips Medical Systems, Bothell, WA, USA) equipped with an L12-5 or L9-3 transducer that allowed the examiner to work in fundamental grayscale and the color Doppler mode. The examiner was blinded to the results of CTA. The grey-scale ultrasound was performed first to examine the location, size, echogenicity, and boundary of the cervical mass. The Shamblin type was defined according to the classification criterion mentioned above (7, 8). The CDU was then used to classify the vascularization as absent, minimal, moderate or marked according to previous study (14). The following were recorded: the inner diameter, peak systolic velocity (PSV) and resistance index (RI) of common, internal carotid artery (ICA) and external carotid artery (ECA). Also noted were the feeding vessels of the tumor from either ICA or ECA when detected.

Next, CEUS was performed under the same installment with a mechanical index of 0.05–0.08, compression of 33–35 and dynamic spatial reconstructor (DSR) middle setting. Sulfur hexafluoride (SonoVue^®^) was used as the ultrasound contrast agent and was dissolved in 5ml saline. Patients were maintained in a supine position, with a 20- or 22-gauge peripheral intravenous cannula, an intravenous bolus of 1.2ml was injected into the median cubital veins within less than 2 seconds, followed by a 5ml saline flush. Meanwhile, the timer on the US machine was started, and the imaging plane was kept as stable as possible. The neck mass was scanned continuously for the 120-second analysis, and a video clip was digitally stored as raw data on a personal computer–based workstation connected to the US unit *via* a standard Ethernet link. The patients were asked not to swallow and to breathe superficially to prevent severe motion of the carotid structure during the scanning.

### Image Post-Processing

The video clips were analyzed on a post-processing workstation (The QLAB quantification software, Philips). The pattern of the contrast enhancement within the CBT lesion was assessed. Vascularity was determined to be absent, minimal, moderate or marked based on the amount of contrast enhancement visualized during the start of the artery phase. In general, enhancement could be observed less than 25% of the lesion area was considered minimal. Between 25% and 50% was considered moderate, and more than 50%, marked. The feeding vessels of the lesion areas deriving from the ICA or ECA and into the tumor were recorded. Time–intensity curves (TICs) were obtained within regions-of-interests (ROIs). ROIs were placed within the areas with the quickest contrast enhancement dynamic since less or no contrast enhancement was suggestive of scar or necrotic tissue. The contrast enhancement feature was depicted in two phases: phase I was the artery phase for contrast enhancement (< 30 sec), and phase II was the wash-out phase for venous and late enhancement (31- sec). The wash in time (WIT), time to peak intensity, time from peak to ½ (wash out time, WOT), and peak intensity were recorded. The area under the curve (AUC) was produced by the software. A 120-second-long analysis was run for all CBT lesions.

### Surgery

Surgical excision of CBTs was described in detail in a previous study (15). Of the 20 CBT patients enrolled in the study, 14 underwent surgical therapy and no surgical mortality or severe morbidity was observed. Other patients didn’t received surgery because of small lesions suitable for waiting and scanning strategy, lesions with systemic metastasis or patients’ refusal to be operated due to concerns about surgical complications.

### Statistical Analysis

Paired-sample tests were used to analyze the difference between grey-scale ultrasound and CEUS for the dimension of CBT lesions. The chi-square test was used for the Shamblin type and feeding vessels of CBT lesions. Independent t-test or Mann-Whitney U test were used to compare the AUCs between different vascularity groups according to the distribution normality. SPSS statistical analysis software (IBM SPSS Statistic version 23, Chicago, IL, USA) was used for statistical significance, which was defined as a level of less than 0.05 for all p-values.

## Results

### Demographics and Tumor Information

**Figure 1:**
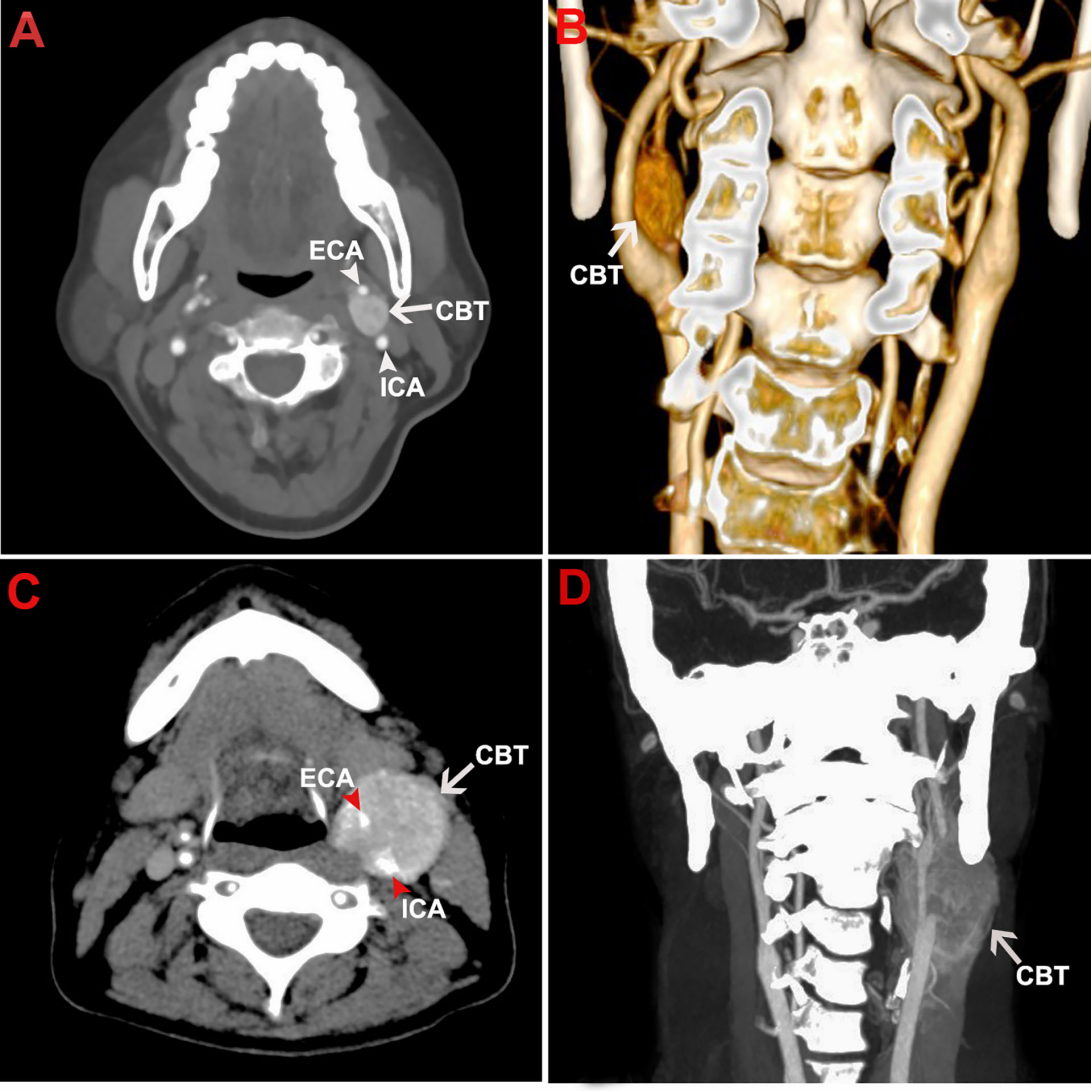
Representative CTA images of CBT. The figure shows the representative CTA images of a Shamblin type I CBT in axial view **(A)** and coronal view **(B)**. CTA images of a Shamblin type III CBT were shown in axial view **(C)** and coronal view **(D)**. The tumor lesions displayed typical radiographic characteristics of CBTs with localization at carotid bifurcation, splaying of carotid arteries, and hypervascularity. No encasement and complete encasement to ICA or ECA were noticed in Shamblin I **(A, B)** and Shamblin III lesions **(C, D)**, respectively. CBT, carotid body tumor; CTA, computed tomography angiography; ICA, internal carotid artery; ECA, external carotid artery.

20 patients (11 male, 9 female) aging from 23 to 63 years old (mean age, 39 years old), had been diagnosed with CBT according to the CTA results (**Figure 1**). Bilateral lesions were identified in 6 patients, generating a total of 26 CBT lesions. Demagraphics and tumor information were shown in **Table 1**. One small lesion (0.7cm x 0.6cm) demonstrated by CTA was not detected by ultrasougraphy and was excluded in following studies. Additionally, two lesions were eliminated from the study of TIC analysis because the two patients moved too much during the recording for the results to be useful, another lesion was excluded from the morphology study because it was too big (7.4 x 10.1cm) to run dimension and Shamblin type measurement. Therefore, a total of 23 and 24 lesions were included in the TIC analysis and morphology study, respectively.

**Table 1:** CBT patient characteristics.

Patient information	numbers
Gender	
Male	11 (55%)
Female	9 (45%)
Mean age (years)	39 (23–63)^a^
Location	
Left	8 (30.8%)
Right	6 (23.1%)
Both	6 (23.1%)
Lesion number	26
Family history	4
CDU	25
CEUS	25
Surgical treatment (n)	14

CBT, carotid body tumor; CDU, color Doppler ultrasound; CEUS, contrast-enhanced ultrasound.

a Age range.

Numbers in the parentheses are percentages.

### Contrast-Enhancement Features and Time–Intensity Curve Analysis

**Figure 2:**
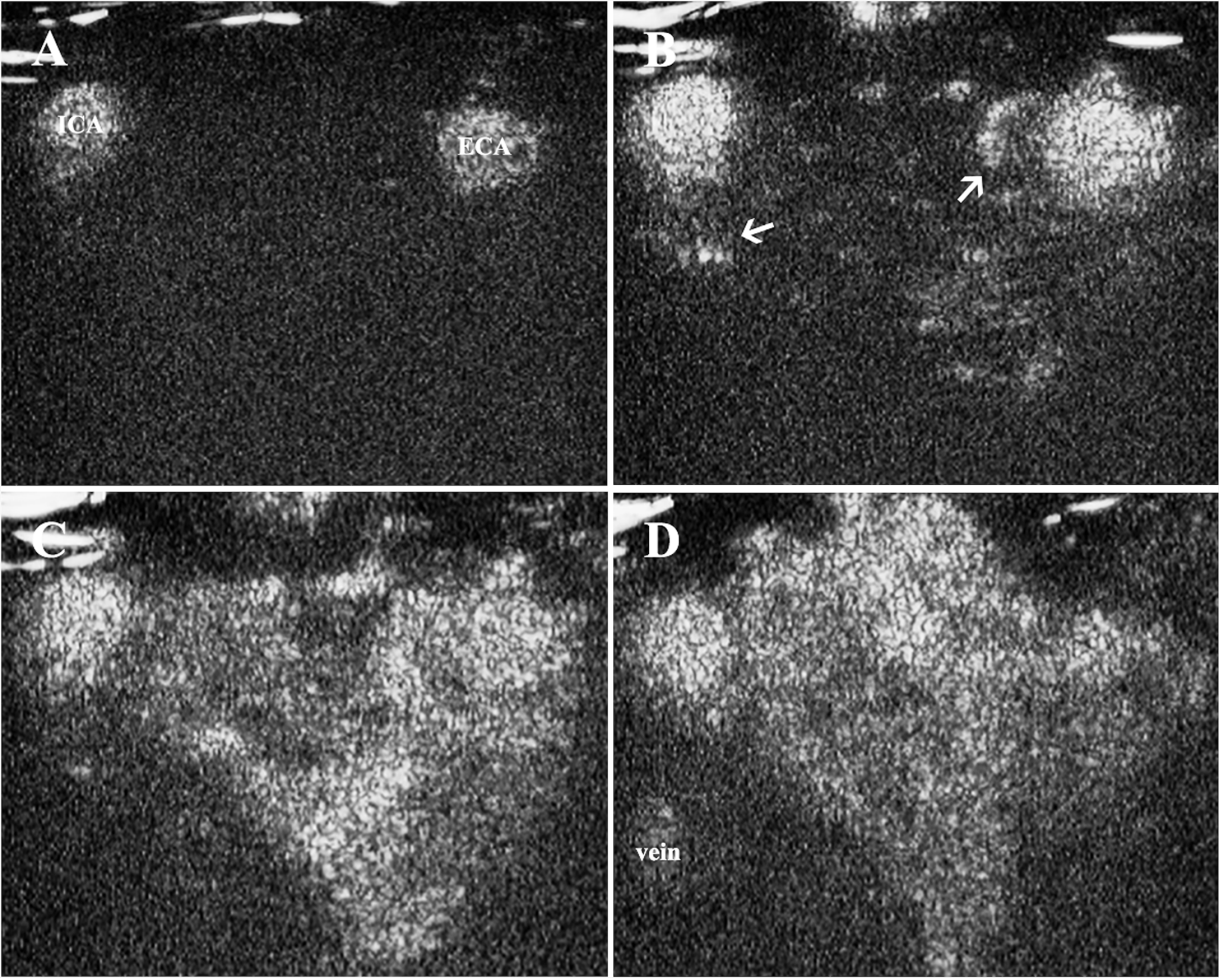
Contrast enhancement in CBT lesion area. **(A)** Contrast enhancement was firstly observed in carotid arteries, **(B)** followed by the enhancement of tumor feeding vessels deriving from ECA and ICA (thin arrows) 10 s after the injection of the contrast agent. **(C)** The contrast agents then diffused rapidly from feeding vessels and exhibited progressing enhancement in the solid area. **(D)** The signal intensity decreased within the carotid arteries and solid area of the tumor, and the enhancement could be observed in the jugular veins. CBT, carotid body tumor; ICA, internal carotid artery; ECA, external carotid artery.

The contrast enhancement was observed in the CBT lesion areas very soon after the injection of microbubbles. After the carotid arteries were enhanced (**Figures 2A, B**), the tumor feeding vessels could be visualized as deriving from either ECA or ICA, or both of them (**Figure 2B**). The contrast agents then diffused rapidly from feeding vessels and exhibited progressing enhancement in the lesion areas during the artery phase (**Figure 2C**). During the wash-out phase, the signal intensity decreased first within the carotid arteries, followed by the enhancement in the jugular veins and the gradual wash-out in the solid area (**Figure 2D**).

**Figure 3:**
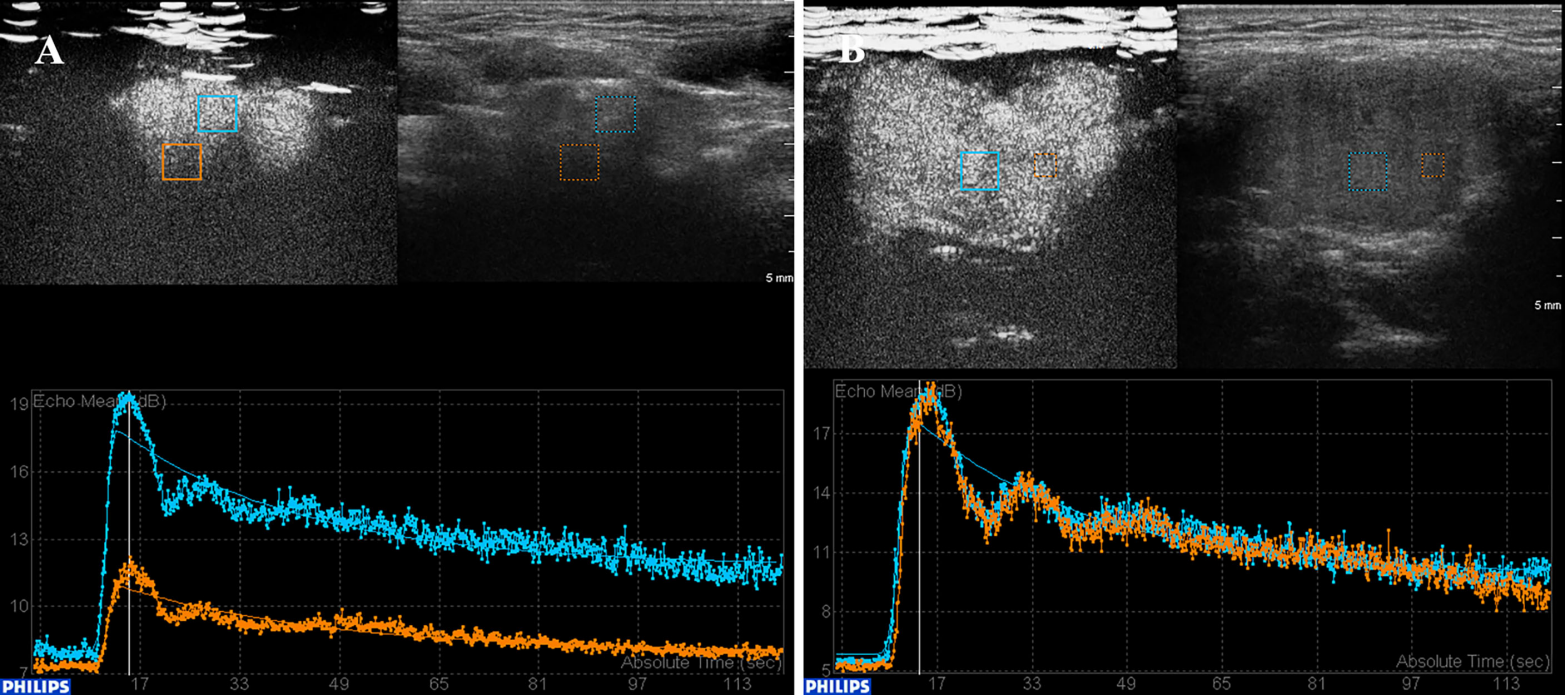
TIC analysis of CBT lesions with heterogeneous and homogeneous enhancement. **(A)** TIC analysis revealed a heterogeneously enhanced lesion. The lesion showed a fast wash-in, slow wash-out, with high peak intensity enhancement pattern. **(B)** A homogeneous enhancement lesion area. CBT, carotid body tumor; TIC, time–intensity curve.

TIC analysis was used to investigate quantitatively the role of CEUS in characterizing the CBTs. The analysis confirmed 11 and 12 CBT lesion areas as homogeneously and heterogeneously enhanced, respectvely. The CBT lesion areas presented a fast wash-in [wash-in-time (WIT): 3.00 ± 1.10 s, mean ± SD] and slow wash-out [wash-out time (WOT): 58.79 ± 24.21s, mean ± SD] pattern (**Figures 3A, B**), with a high peak intensity as 10.56 ± 1.57dB (mean ± SD). In our study, the gross enhancement during the time-course that presented as AUC ranged widely from 370.59 mm^2^ to 978.17 mm^2^, with an average of 669.68 ± 143.46 mm^2^ (mean ± SD). No significant correlation was found between TIC parameters and patients’ gender, lesion dimension, and Shamblin type.

### Morphology Study

**Figure 4:**
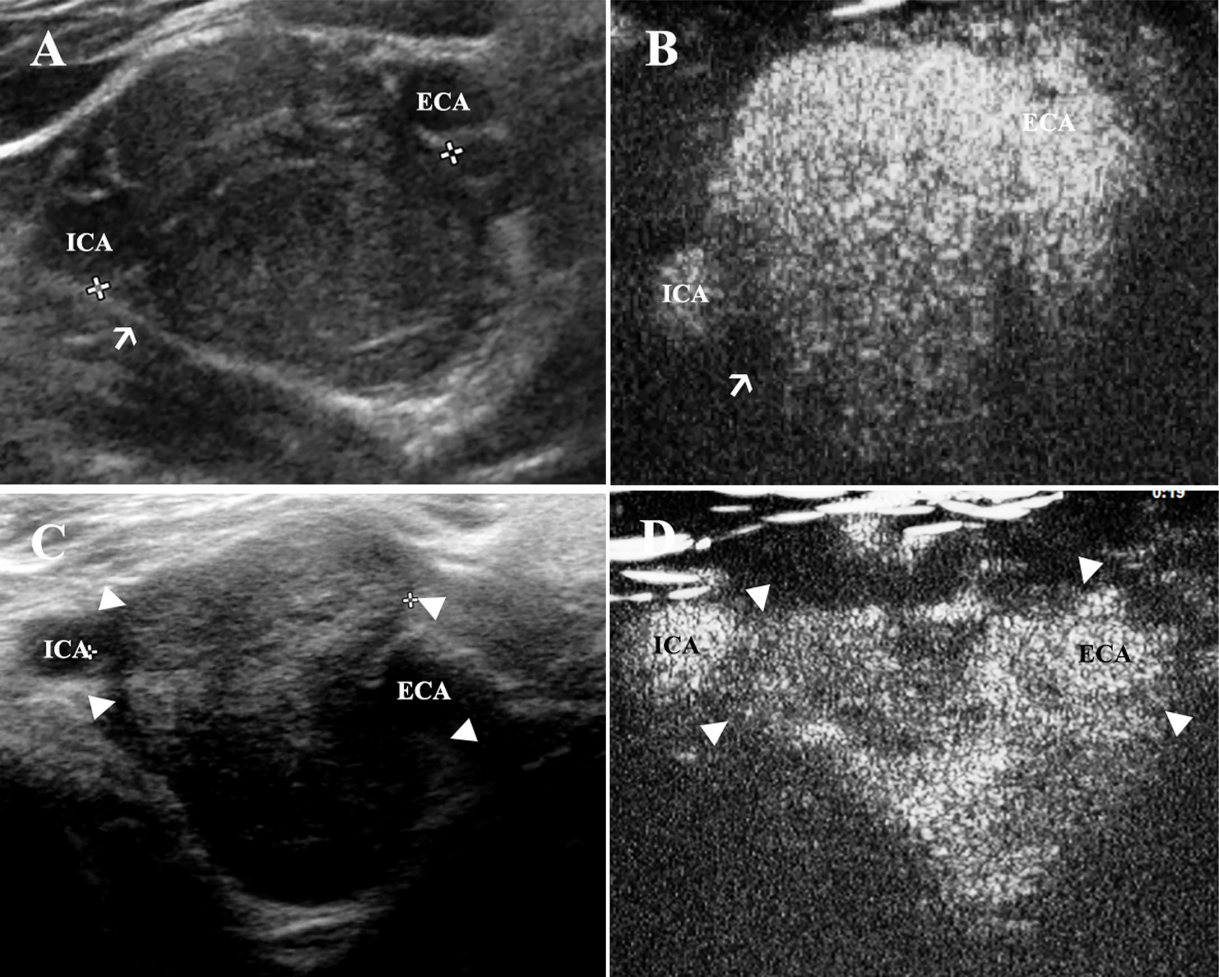
CEUS is superior to assess the morphology of CBT lesions in comparison to gray-scale ultrasound. **(A)** CBT lesions presented as oval-shaped and hypoechoic masses, which led to splaying of the carotid bifurcation. The adventitia of carotid vessels could be observed clearly on gray-scale ultrasound (thin arrow). **(B)** However, the adventitia (thin arrow) could hardly be visualized in CEUS during the artery phase. **(C)** A very small part of ICA was encased by the lesion area. The margin (triangles) of the tumor around ECA could not be seen clearly, and a Shamblin type II was identified using grey-scale ultrasound. **(D)** The margin of the tumor (triangles) could be better observed using CEUS and a Shamblin type III was reclassified. CBT, carotid body tumor; CEUS, contrast-enhanced ultrasonography; ICA, internal carotid artery; ECA, external carotid artery.

Gray-scale ultrasound presented all CBT lesions as oval-shaped and hypoechoic masses that led to the splaying of the bifurcation and separation of the ICA and ECA (**Figure 4A**). The adventitia of the carotid vessels that encased CBT lesion areas could be observed clearly on gray-scale ultrasound (**Figure 4A**). In contrast, the adventitia did not enhance intensely; therefore, the real margins of the lesion could be better visualized in CEUS (**Figure 4B**). As shown in **Table 2**, CDU and CEUS were not found to be significantly different in the measurement of the dimension of lesion areas. The mean maximum diameter of the lesions detected by gray-scale ultrasound was 2.46 ± 0.92 × 3.02 ± 1.23 cm (mean ± SD), and the CEUS result was 2.51 ± 0.92 × 2.85 ± 1.03 cm (mean ± SD) (**Table 2**). The Shamblin type defined by gray-scale ultrasound and CEUS is presented in **Table 2**. While there was a correlation between gray-scale ultrasound and CEUS in identifying type II CBT lesions, the CEUS technique was superior to gray-scale ultrasound for detecting type I and III CBT lesions (**Figures 4C, D**). CEUS reclassified 2 lesions from type I to type II and upgraded 4 lesions from type II to type III. The Pearson chi-square test, which was used to test the differences in the shambling types defined by CDU and CEUS, showed a significant difference between these two modalities (χ^2 ^= 17.389, p=0.002).

**Table 2:** Morphology of CBT lesions (n = 24).

Lesion areas	Gray-scale	CEUS
size (cm)		
Anterior–posterior	2.46 ± 0.92	2.51 ± 0.92^a^
Left–right	3.02 ± 1.23	2.85 ± 1.03^b^
Shamblin type^**^		
I	4 (16.7)	2 (8.3)
II	18 (75.0)	16 (66.7)
III	2 (8.3)	6 (25.0)

CBT, carotid body tumor; CEUS, contrast-enhanced ultrasound.

Numbers in parentheses are percentages.

a Paired T-test, t=0.414, p=0.683.

b Paired T-test, t=-1.265, p=0.219.

^**^Pearson chi-square test, χ^2 ^= 17.389, p=0.002.

### Vascularity and Feeding Vessels

**Figure 5:**
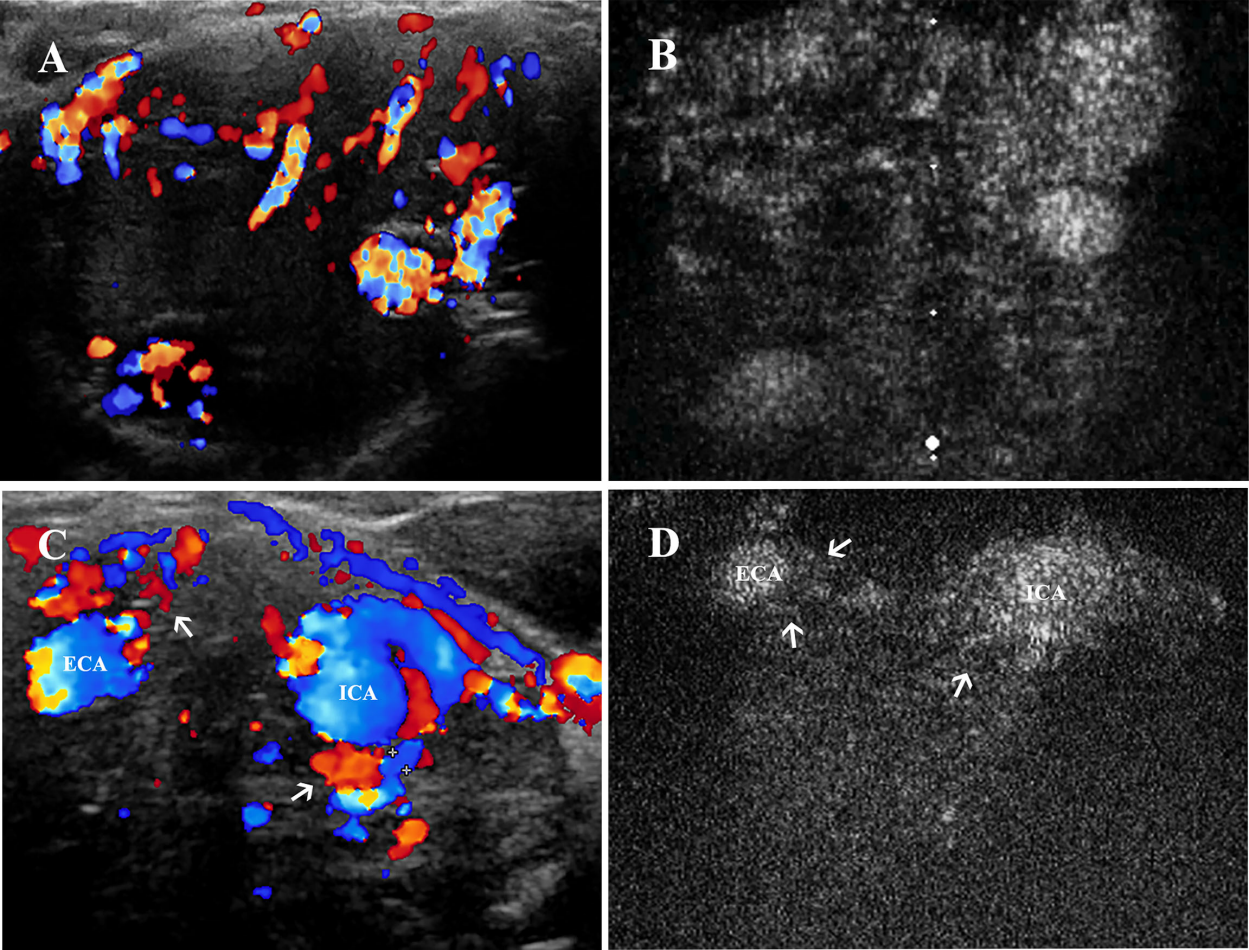
Vascularity and feeding vessels of CBT lesions. **(A)** CDU showed a hypervascularity and hypoechoic mass located at carotid bifurcation, and **(B)** robust signal intensity could be seen 1–2 s after the enhancement of carotid arteries on CEUS. **(C)** Feeding vessels (thin arrows) of another CBT lesion could be observed deriving from both ECA and ICA on CDU and **(D)** on CEUS. CBT, carotid body tumor; CEUS, contrast-enhanced ultrasonography; CDU, color Doppler ultrasound; ICA, internal carotid artery; ECA, external carotid artery.

CDU revealed the hypervascularity feature of CBT lesions located at the carotid bifurcation (**Figure 5A**), while CEUS showed robust signal intensity during the artery phase of contrast enhancement (**Figure 5B**). **Table 3** illustrated the vascularity of the CBTs detected by CDU and CEUS. Moderate and marked vascularities accounted for 80% of the lesions in CDU and 100% in CEUS. We compared the vascularity grades obtained by CDU and CEUS with AUC to better explore the value of CEUS in evaluating the vascularity of CBT lesions. For CDU, minimal vascularity lesions had an AUC as 608.89 ± 78.58 mm^2^ (mean ± SD). The Moderate group and marked group had an AUC as 637.67 ± 160.36 mm^2^ and 722.81 ± 139.96 mm^2^ (mean ± SD), but no significant difference was found between groups. In comparison with CDU, CEUS classified vascularity grades that correlated better with the AUC values. CEUS also detected a significant difference in AUC between moderate and marked groups (563.33 ± 102.63 vs. 707.22 ± 138.81, t=-2.311, p=0.031.).

**Table 3:** Vascularity feature of CBTs.

	CDU	CEUS
**Classification (n=25)**		
Absent	0	0
Minimal	5 (20%)	0
Moderate	9 (36%)	8 (32%)
Marked	11 (44%)	17 (68%)
**AUC (n=23, mm^2^)**		
Absent	N/A	N/A
Minimal	608.89 ± 78.58 (n=4)	N/A
Moderate	637.67 ± 160.36 (n=9)^a^	563.33 ± 102.63 (n=6)
Marked	722.81 ± 139.96 (n=10)^b^	707.22 ± 138.81 (n=17)^c^
**Feeding vessels (n=25)** ^**^		
ICA	0	0
ECA	6 (24%)	9 (36%)
Both	19 (76%)	13 (52%)
Undetermined	0	3 (12%)

CBT, carotid body tumor; CDU, color Doppler ultrasound; CEUS, contrast-enhanced ultrasound; AUC, area under the curve; ICA, internal carotid artery; ECA, external carotid artery; N/A, Not available.

AUC values are presented as mean ± standard deviation.

a Compared minimal and moderate vascularity in CDU, t=-0.335, p=0.744.

b Compared moderate and marked vascularity in CDU, t=-1.236, p=0.233.

c Compared moderate and marked vascularity in CEUS, t=-2.311, p=0.031.

^**^Pearson chi-square test, χ^2 ^= 9.162, p=0.010.

To better investigate the feeding vessels of CBT lesion areas, we assessed all 25 lesion areas by CDU (**Figure 5C**) and CEUS (**Figure 5D**). A significant difference was found (Pearson chi-square test, χ^2 ^= 9.162, p=0.010): CDU was more sensitive in detecting feeding vessels than CEUS (100% vs. 88%). The feeding vessels of CBTs derived from either ECA (24%) or both ICA and ECA (76.0%) were found on CDU. On CEUS, the feeding vessels were visualized originating from ECA (36%) and both carotid arteries (52%); however, CEUS failed to detect the feeding vessels of the three lesions due to the suboptimal location. Notably, two of these 3 feeding vessels could be speculated according to the enhancement pattern on CEUS, and the results were the same as the CDU findings.

## Discussion

With the introduction of ultrasonography contrast agents, CEUS has overcome the limitations of CDU by allowing a dynamic enhancement assessment of lesions similar to CTA and also providing the benefits of a paucity of contrast material side effects (16). The contrast agents of CEUS consist of gas molecules stabilized by lipid or albumin; they do not contain iodine and are extracted from the body through the pulmonary system, thus avoiding exposure to ionizing radiation and iodine contrast-related nephrotoxicity (17). In general, the contrast is very safe to patients, with a low rate of anaphylactoid reactions compared to the other types of contrast agents (18). Although some mild adverse events have been reported with the use of a CEUS contrast agent, such as injection site pain or rash, light headache, chest discomfort, nausea, and hyperventilation (19), the ultrasound contrast is considered the safest agent among other types of contrast media, with a very low frequency of severe adverse events (0.007%–0.0086%) (18, 20), which is lower than that of iodinated media and lower than or similar to that of gadolinium agents (21, 22). The value of CEUS in the evaluation of several tumors and vascular disease has been proven by other studies (10, 23, 24), while its utilization in CBT assessment remains to be investigated.

Our study firstly used CEUS to investigate the contrast enhancement feature of CBTs. We found that CBTs showed a fast wash-in, slow wash-out pattern with high AUC and a peak intensity enhancement signature, with a mean WIT and WOT of 3.00 ± 1.10 s and 58.79 ± 24.21 s (mean ± SD), respectively. Among all TIC parameters, WIT had the most consistent result, which was most likely due to their hypervascularity feature and close connection to carotid arteries. We also compared CEUS to conventional CDU for the evaluation of the lesion morphology and Shamblin type. We found that, first, CEUS was superior to gray-scale ultrasound in evaluating the morphology of lesion areas. It better enhanced and thus visualized the margins of the CBTs, given that the adventitia of carotid vessels in gray-scale ultrasound sometimes interferes with the discrimination of those margins. Besides, CEUS was also found to be more accurate in describing the Shamblin type of CBT lesions, which might contribute to the preoperative preparation and surgical planning of CBTs because a higher Shamblin type has been shown to be correlated with a higher risk of vascular injury and surgical bleeding during a surgical excision of the lesions (25).

In addition, we found that CEUS could better evaluate the vasculature abundance of CBTs in comparison to CDU in that CDU showed no significant difference in the AUC between the different vascularity groups it obtained. The discrepancy between the vascularity grades and enhancement intensity might have resulted from the lower sensitivity to small feeding vessels of CDU. CEUS, in contrast, uses microbubbles that are small enough (∼3 μm) to remain intact when flowing through arterioles and big enough to stay within the vascular compartment without passing through capillary fenestrations (26). Therefore, CEUS is a great tool for the visualization of tumor microvessels using a very small amount of contrast agents (27, 28). The vascularity classified by CEUS was found to correlate more with the AUC values, and a significant difference was also observed between different groups. These findings indicate that CEUS could provide a better evaluation of the vascularity than CDU both qualitatively and quantitatively.

Contrastly, CDU showed a higher sensitivity in the present study as compared to CEUS, in detecting the feeding vessels of CBTs (100% vs. 88%), while other studies had reported the opposite findings (29). It was difficult for both CEUS and CDU to identify the origin of the microvessels when the lesion contained abundant blood vessels. When the feeding vessel was detectable, however, CEUS was superior to CDU in determining the origin of the vessel since it could visualize the dynamic contrast enhancement within the micro-vessels, as revealed by our study (χ^2 ^= 9.162, p=0.010). Although superior, CEUS image acquisition relied on two-dimensional (2D) long and transverse section imaging and displayed the blood feeding vessels in a single plane. Our study concluded that 2D-CEUS could not fully capture the vascular spatial heterogeneity if the lesion was detected in a suboptimal position, a conclusion that is in accordance with previous studies (30). Neverthless, since preoperative embolization or early ligation of the feeding vessels prior to the resection of the tumor could benefit from early detection of the origins of feeding vessels, CEUS could potentially contribute to a bloodless operative field and therefore reduce the operative morbidity, due to its superiority in identifying the origin of feeding vessels (31, 32).

Our study had several limitations. First, there was no reference tissue for CBT lesions owing to their particular location, and the lack of the reference limited the characteristic description of the contrast enhancement pattern as in other solid organs. Second, 2D-CEUS could not fully capture the morphology and vascularity spatial heterogeneity when the lesion was detected in a suboptimal position and the largest dimension could not be acquired within the plane with the highest Shamblin grade sometimes. Neverthless, this is, to the best of our knowledge, the first prospective comparative study that investigated the performance of CEUS on the evaluation of CBTs, and our findings might give new perspectives on preoperative evaluation of the lesions when computed tomography or magnetic resonance imaging modalities are unsuitable or not available. Based on our findings, CEUS is superior to CDU in evaluating the morphology, Shamblin typing, vascularity, and origins of the feeding vessels of CBT lesions. More importantly, the advantages of CEUS are especially in monitoring the evolution of the CBTs including the location, size, growth rate, vascular encasement, and dynamic evaluation of the vasculature with a low cost, practicality, and lack of ionizing radiation. Beyond that, CEUS might show advantages in the differential diagnosis of CBTs. For example, our preliminary studies showed a distinct enhancement pattern of schwannomas, another neoplasm located at carotid bifurcation, as compared to CBTs, which exhibited a ring-like enhancement pattern and revealed a slower wash-in, faster wash-out pattern with lower peak intensity in TIC analysis (**Supplementary Figures 1**, **2**). Future studies might be conducted to complement the current findings by introducing the 3D analysis of CEUS (30) and including adjacent tissues such as carotid arteries as reference. Besides, future comparative studies with larger sample size will help to validate the advantage of CEUS in the differential diagnosis between CBTs and other neoplasms at carotid bifurcation including schwannomas.

## Conclusion

TIC analysis showed that CBT displayed consistently a fast wash in, slow wash-out pattern with high AUC. Both CDU and CEUS are appropriate to use in evaluating the morphology and vascularity of CBTs. In comparison with CDU, CEUS can better assess the Shamblin type of the lesion areas, especially for Shamblin I and III types. CEUS can also define the vascularity grades that are correlated better with TIC values. CEUS is superior to CDU in visualizing the origin of feeding vessels due to its excellent temporal resolution, but it has lower sensitivity than CDU.

## Data Availability Statement

The raw data supporting the conclusions of this article will be made available by the authors, without undue reservation.

## Ethics Statement

The studies involving human participants were reviewed and approved by the institutional review board of Peking Union Medical College Hospital. The patients/participants provided their written informed consent to participate in this study.

## Author Contributions

GG, XZ, and JS: conceptualization, methodology, formal analysis, investigation, data curation, manuscript drafting. SG, QG, JS, and BL: investigation, data curation and manuscript editing. BZ: conceptualization, methodology, supervision, formal analysis, manuscript review and editing. YZ: conceptualization, methodology, manuscript review and editing, supervision and funding acquisition. All authors contributed to the article and approved the submitted version.

## Funding

This work was supported by grant from the Major Research Program of Natural Science Foundation of China (51890892), The Natural Science Foundation of China (82070492 & 81770481), The Non-profit Central Research Institute Fund of Chinese Academy of Medical Sciences (2021-JKCS-027), The Chinese Academy of Medical Sciences Innovation Fund for Medical Sciences (Grant No.CIFMS2021-I2M-1-016) and The CAMS Innovation fund for Medical Science (CIFMS, Grant No.2021-I2M-C&T-A-006).

## Conflict of Interest

The authors declare that the research was conducted in the absence of any commercial or financial relationships that could be construed as a potential conflict of interest.

## Publisher’s Note

All claims expressed in this article are solely those of the authors and do not necessarily represent those of their affiliated organizations, or those of the publisher, the editors and the reviewers. Any product that may be evaluated in this article, or claim that may be made by its manufacturer, is not guaranteed or endorsed by the publisher.

## Supplementary Material

Supplementary Figure 1 ring-like enhancement observed in a schwannoma lesion. The epineurium (thin arrow) of schwannoma lesion was clearly enhanced during the artery phase and exhibited a ring-like enhancement pattern.

Supplementary Figure 2 TIC analysis of schwannoma lesions. Schwannomas showed a slow wash-in, fast wash-out and low peak intensity pattern in TIC analysis.

## Abbreviations

CBT, carotid body tumor; CDU, color Doppler ultrasound; CEUS, contrast-enhanced ultrasonography; TIC, time–intensity curve; WIT, wash-in time WOT, wash out time (time from peak to ½); ICA, internal carotid artery; ECA, external carotid artery.
