# Supplementary figures and images for: Comparison of Contrast-Enhanced Ultrasonography to Color Doppler Ultrasound in Evaluation of Carotid Body Tumors
Source: Front Oncol. 2022 Apr 11;12:872890. doi: 10.3389/fonc.2022.872890 (PMC9035876; doi:10.3389/fonc.2022.872890)

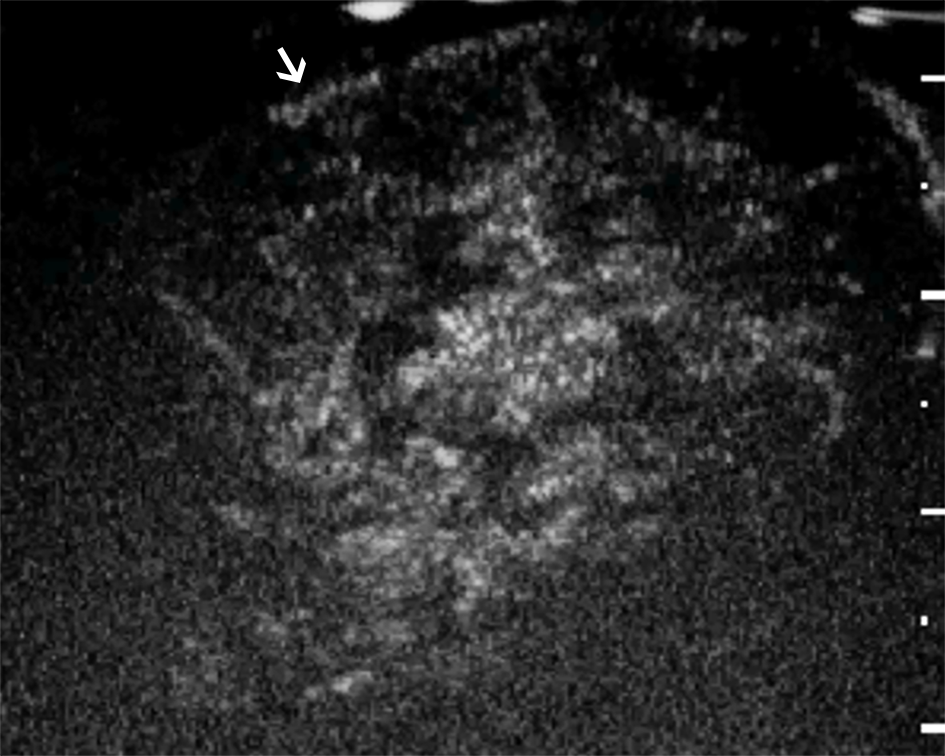

Supplement: Supplementary Figure 1 — ring-like enhancement observed in a schwannoma lesion. The epineurium (thin arrow) of schwannoma lesion was clearly enhanced during the artery phase and exhibited a ring-like enhancement pattern. [file Image_1.tif]

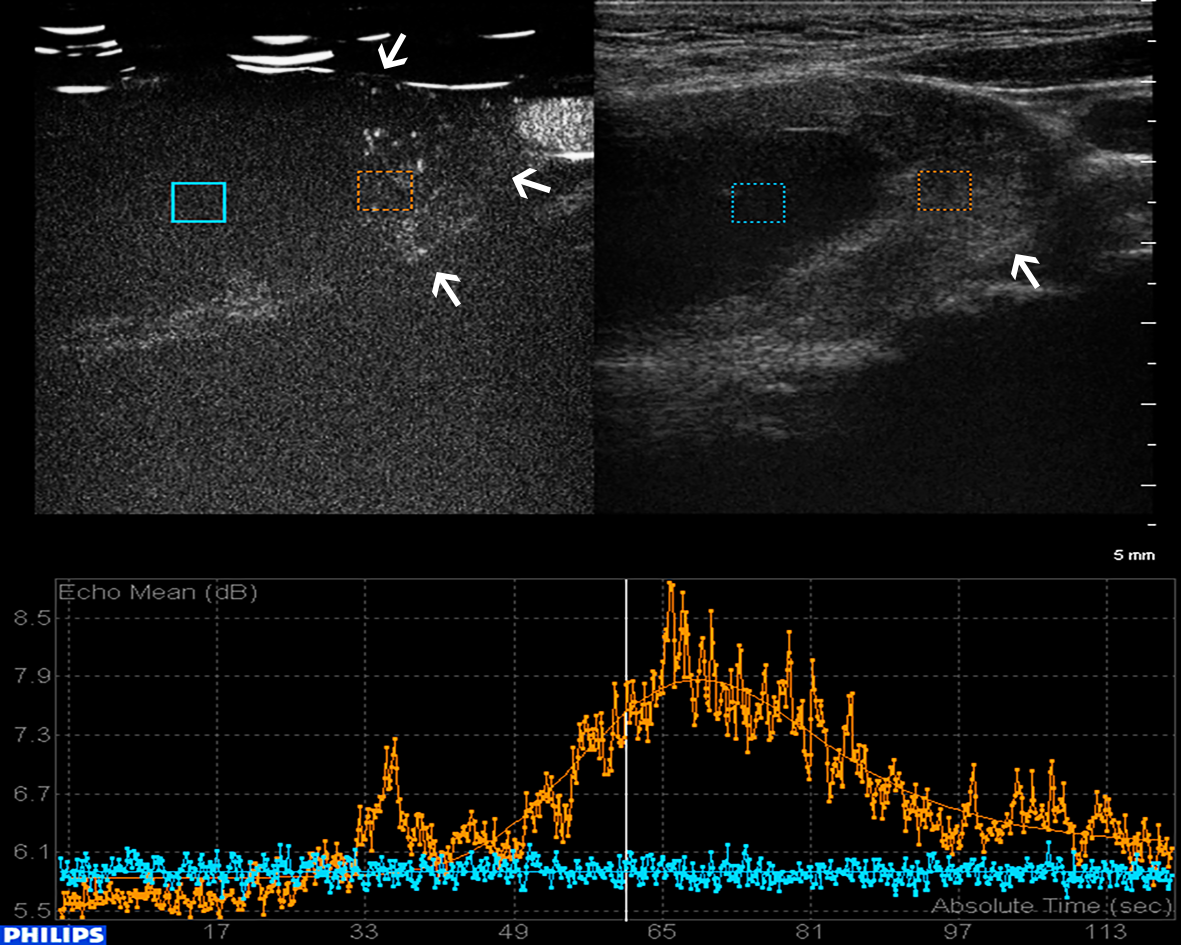

Supplement: Supplementary Figure 2 — TIC analysis of schwannoma lesions. Schwannomas showed a slow wash-in, fast wash-out and low peak intensity pattern in TIC analysis. [file Image_2.tif]
